# The spatial configuration of local climate zones reveals effects on wayfinding in human walking

**DOI:** 10.1371/journal.pone.0289780

**Published:** 2023-09-08

**Authors:** Ping Yu Fan, Kwok Pan Chun, Mou Leong Tan, Daphne Ngar-Yin Mah, Ana Mijic, Graham Strickert, Omer Yetemen

**Affiliations:** 1 Department of Urban Planning and Design, The University of Hong Kong, Hong Kong S. A. R., China; 2 Department of Geography and Environmental Management, University of the West of England, Bristol, United Kingdom; 3 GeoInformatic Unit, Geography Section, School of Humanities, Universiti Sains Malaysia, Gelugor, Penang, Malaysia; 4 School of Geography, Nanjing Normal University, Nanjing, China; 5 Department of Geography, Hong Kong Baptist University, Hong Kong S. A. R., China; 6 Department of Civil and Environmental Engineering, Imperial College London, London, United Kingdom; 7 School of Environment and Sustainability, University of Saskatchewan, Saskatoon, Canada; 8 Eurasia Institute of Earth Science, Istanbul Technical University, Istanbul, Turkey; Huazhong University of Science and Technology, CHINA

## Abstract

The importance of easy wayfinding in complex urban settings has been recognized in spatial planning. Empirical measurement and explicit representation of wayfinding, however, have been limited in deciding spatial configurations. Our study proposed and tested an approach to improving wayfinding by incorporating spatial analysis of urban forms in the Guangdong-Hong Kong-Macau Great Bay Area in China. Wayfinding was measured by an indicator of intelligibility using spatial design network analysis. Urban spatial configurations were quantified using landscape metrics to describe the spatial layouts of local climate zones (LCZs) as standardized urban forms. The statistical analysis demonstrated the significant associations between urban spatial configurations and wayfinding. These findings suggested, to improve wayfinding, 1) dispersing LCZ 1 (compact high-rise) and LCZ 2 (compact mid-rise) and 2) agglomerating LCZ 3 (compact low-rise), LCZ 5 (open mid-rise), LCZ 6 (open low-rise), and LCZ 9 (sparsely built). To our knowledge, this study is the first to incorporate the LCZ classification system into the wayfinding field, clearly providing empirically-supported solutions for dispersing and agglomerating spatial configurations. Our findings also provide insights for human-centered spatial planning by spatial co-development at local, urban, and regional levels.

## 1. Introduction

Wayfinding is rarely explicitly recognized and presented in determining the agglomerated or dispersed urban spatial layouts [1], despite its importance in facilitating human moving behavior and improving the human travel experience. Wayfinding is the process by which people locate themselves, find their way, and then move from one place to another by perceiving and organizing the surrounding environment [2]. Improving wayfinding encourages walking behavior over vehicle navigation because people find it easier to position themselves in urban environment [3]. The exclusion of wayfinding from spatial planning increases the likelihood of people being lost in complex urban settings, resulting in psychological discomfort. Therefore, improving wayfinding could address a question of ‘where am I?’, which contributes to a more comfortable movement experience by reconfiguring urban spaces.

Wayfinding has been frequently investigated in environmental psychology using a spatial cognition perspective. Spatial cognition is broadly defined as the understanding of the relationships between the self and their surrounding environments and objects [4]. Moving through space, the various city images are created in the human mind due to different spatial knowledge acquired from the process of spatial cognition [5]. These city images can be the cognitive representations of the external environment to guide human wayfinding in movement. The more spatial information people can cognize from the surroundings while navigating, the easier it is for them to achieve wayfinding. Based on the links between spatial cognition and wayfinding, environmental psychologists pay close attention to the physical characteristics of urban spaces for facilitating easy wayfinding [4]. Kevin Lynch was one of the pioneers who incorporated spatial cognition into urban planning, based on people-space interactions [6]. He proposed a concept of ‘legibility’ in the book—the image of the city [7]—to suggest the effects of urban spatial characteristics on wayfinding. Following Kevin Lynch, increasingly researchers combined the wayfinding related to spatial cognition and urban spatial planning with the development of advanced analytical tools.

Space syntax is a technique for quantifying wayfinding by modelling urban spatial characteristics, based on a premise that urban spaces are not the background of society, but rather that spatial and social attributes are intrinsically linked [8]. It implies the validity of measuring wayfinding in human movement using physically spatial characteristics. Moreover, space syntax also highlighted the ‘spatial configuration’ concept that describes the relationships between two spaces by taking into account their relationships with others [9]. It means that space syntax, compared with Kevin Lynch’s works, further provides a network analysis perspective. The roles of spatial configurations in the human movement have long been recognized [10, 11]. For example [12, 13], found that street spatial structure shaped human moving behavior. [14] even directly identified spatial configuration as the determinant of wayfinding. Spatial configurations tend to be heterogeneous across locations, for example, shown as either dispersed or agglomerated patterns. Such heterogeneity may result in incoherent spatial characteristics as well as poor wayfinding ability in human movement. We thus claim that space syntax has the potential to extend the conventionally spatial cognition-based wayfinding in environmental psychology to spatial characteristics-based wayfinding in the urban planning field. However, the space syntax technique describes urban spatial characteristics by simplifying physical spatial relationships into street networks, without taking into account urban data, such as land use and built-up structure [15]. In this way, wayfinding measurement based on space syntax technique has limited implications for and relevance to the implementation of spatial planning strategies.

One approach that may increase the relevance of space syntax-based wayfinding in spatial planning is to incorporate urban form considerations. This could be achieved by measuring the spatial configuration of urban spaces using landscape metrics. Landscape metrics are algorithms that use numeric values to quantify urban spatial characteristics, such as the distribution of land-cover types relative to others [16]. Land-cover type is the most common category for landscape metrics quantification in spatial configuration analysis [17, 18]. However, other than land-cover type, land surface structure—the spatial configuration of various buildings—is often ignored in spatial analysis of urban form. This limitation can be addressed by using the local climate zone (LCZ) classification system that provides ten standardized built types (LCZ1—LCZ10) and seven land cover types (LCZ A—LCZ G) [19]. The LCZ classification provides more details about the urban environment, such as building density and heights, than simply using “urban” or “impervious surface” which is the case in conventional land use classifications. The LCZ classification has been widely used in urban climate studies to represent local microclimates [20]. For example, some studies linked the configuration metrics using LCZ types with air quality [21] and surface temperature [22]. Therefore, our study can be the first attempt to extend the LCZ classification system beyond urban climate studies to the wayfinding field in spatial planning. Quantifying landscape metrics among different LCZ types allows for demonstrating spatial configurations of both surface structures in terms of built-up types and surface covers in terms of land cover types. As a result, incorporating LCZ classification system and landscape metrics-based spatial configuration analysis provides opportunities for modifying land covers and built-up areas for improving wayfinding.

By combining space syntax-based wayfinding and LCZ-based landscape metrics quantification of urban form, this study tested the hypothesis that spatial configurations (e.g., agglomeration or separation) influence wayfinding via LCZ reconfiguration. Our three specific objectives are: 1) quantifying wayfinding using space syntax technique and urban forms using landscape metrics of LCZ types; 2) identifying possible statistical relationships between quantitative wayfinding and LCZ-based urban forms; 3) suggesting reconfiguration strategies of LCZ types for improving wayfinding. This study is structured into five parts. The data, methods, and technical details of measuring wayfinding, mapping LCZ types, and quantifying landscape metrics are described in Section 2. Section 3 delineates the empirical results of heterogeneous wayfinding among cities, quantitative spatial configurations of LCZ types, and their statistical correlations. The implications for wayfinding measurements and urban spatial modifications are discussed in Section 4. Section 5 presents the study conclusions.

## 2. Data and methods

### 2.1 Analytical framework

**Fig 1:**
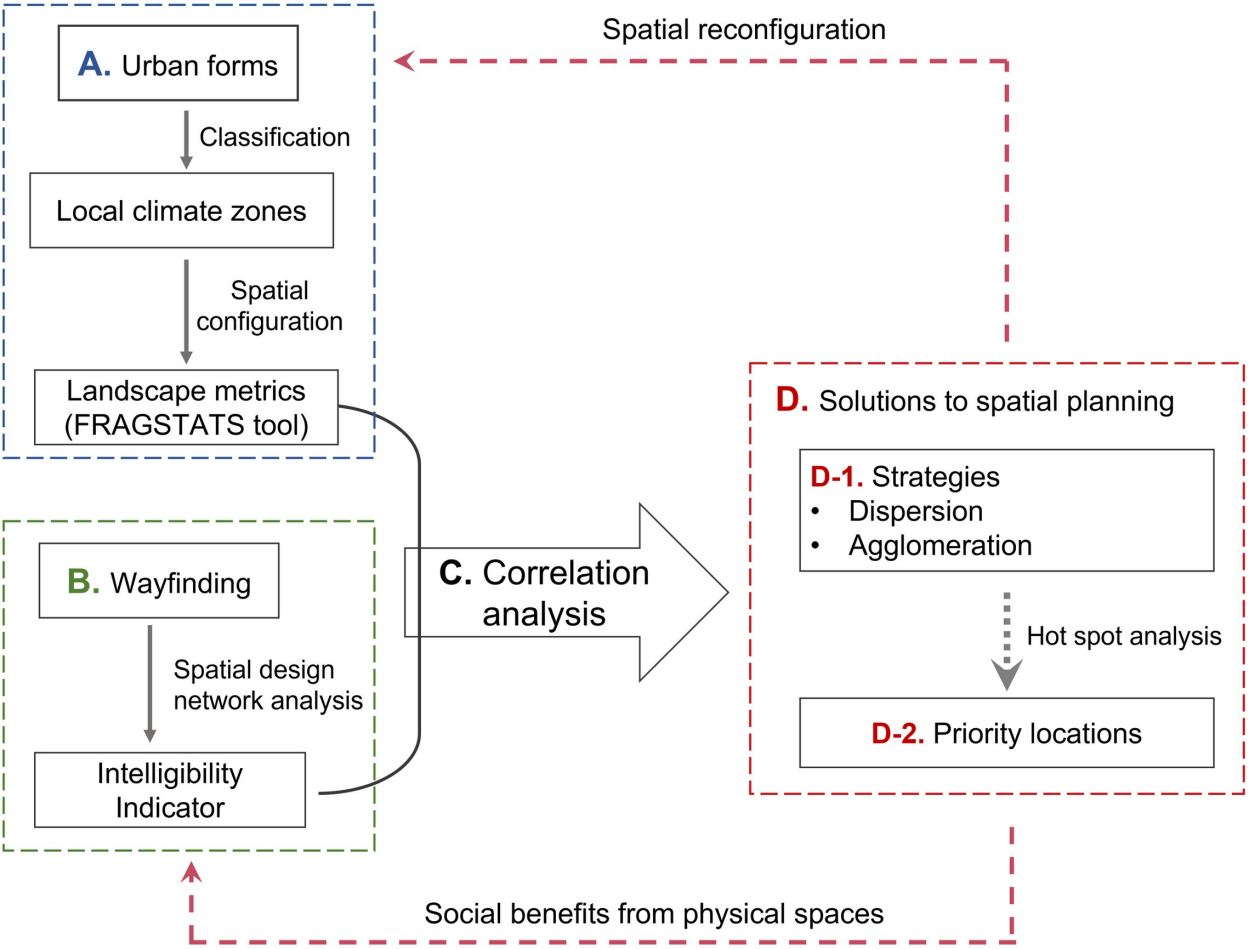
Analytical framework.

The analytical framework for revealing the effects of urban spatial configurations on wayfinding is depicted in Fig 1, presented in four parts. Firstly, to extract spatial characteristic information, the local climate zone (LCZ) classification was used to categorize urban forms and their spatial configurations were quantified by landscape metrics using the FRAGSTATS tool. The values of landscape metrics for each LCZ type were used as proxies for quantitative urban spatial configurations. Moreover, to describe the wayfinding in current urban forms, an intelligibility indicator derived from the space syntax was used to measure wayfinding objectively using spatial design network analysis. The value of the intelligibility indicator was used to represent people’s ability to navigate through a city. After the quantifications of both urban spatial configuration and wayfinding, the third step in this analytical framework is to capture their quantitative relationships. Correlation analysis was thus used to identify the positive or negative relationships between landscape metrics of LCZs and wayfinding. Developing spatial reconfigurations strategies based on correlation results was the final step, allowing planners to decide whether dispersed or agglomerated urban forms were better for people’s wayfinding. Positive correlations indicate a potential improvement in wayfinding by increasing the values of landscape metrics. Additionally, using a hot spot analysis, the spatial clusters of landscape metrics were demonstrated to determine the priority locations for implementing those suggested strategies. The strategies and the locations for spatial reconfiguration were the major outputs of this study.

### 2.2 Case study

**Fig 2:**
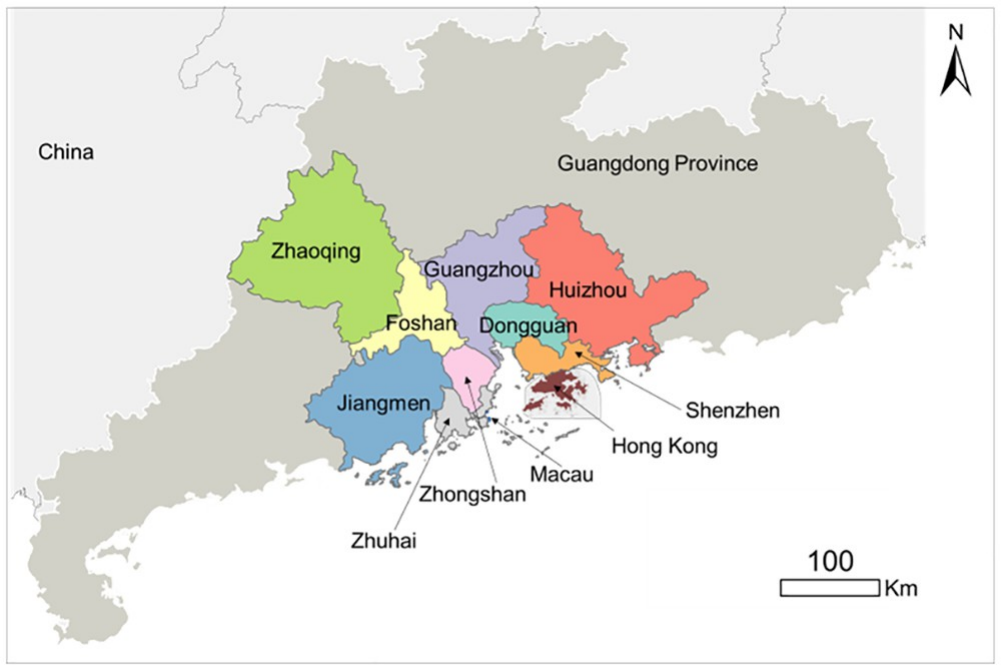
The location of the Guangdong-Hong Kong-Macau Greater Bay Area (GBA).

The analytical framework is tested using the Guangdong-Hong Kong-Macau Greater Bay Area (GBA) as a case study. The GBA is an emerging initiative for rapid regional development in China. Located in southern China, the GBA includes nine cities in the Guangdong Province and two Special Administrative Regions (Hong Kong and Macau) (Fig 2). As a megacity region, the GBA is densely populated with over 70 million people and accounts for around 13% of China’s gross domestic product (GDP). Well-developed street networks for regional integration create strong spatial connections among cities in the GBA [23]. Also, significant changes in land use and cover occurred [24]. Land resource development creates diverse local spatial configurations, which may influence social function and well-being, such as wayfinding. Understanding whether and how the heterogeneous spatial characteristics throughout the GBA affect wayfinding helps to provide a pleasant walking experience for people.

### 2.3 Wayfinding measurements

#### 2.3.1 Intelligibility as the proxy for wayfinding

In space syntax context, wayfinding is quantified by an indicator of intelligibility. Intelligibility, measured as the numeric value, refers to the level of coherence between small- and large-scale urban spatial configuration [25], that is, the extent to which large-scale spatial characteristics of a city can be predicted by small-scale spatial features. Spaces with high intelligibility values have more similar and coherent spatial configurations between local and city levels. People are thus easier orient themselves in a complex built environment by predicting large-scale spatial configurations based on small-scale spatial information within their viewshed [8]. [26] reported the first study to relate wayfinding directly with intelligibility metrics following the space syntax theory. After that, there have been numerous applications of intelligibility based on the space syntax theory in indicating wayfinding in human movement [27, 28].

#### 2.3.2 Quantifying intelligibility indicator using spatial design network analysis

Intelligibility quantification was conducted using spatial design network analysis (sDNA) (https://sdna.cardiff.ac.uk/sdna/). Developed from the space syntax approach, sDNA is an ArcGIS/QGIS/AutoCAD plug-in or Python/command-line tool for analyzing the urban network spatial patterns by simplifying the complex urban spaces into street network representations [29, 30].

To perform the sDNA, street vector layer data was extracted from OpenStreetMap (OSM) in the QGIS software. OSM is an open-source project to collect and provide free geographical information around the world [31], which has been fairly accurate currently. Around 80% of the highway objects overlap those in the real world [32]. Studies also showed that OSM has high quality when compared to authoritative datasets [33, 34]. The roads derived from OSM are categorized into seven classes based on their social function and importance, including ‘Motorway’, ‘Trunk’, ‘Primary’, ‘Secondary’, ‘Tertiary’, ‘Unclassified’, and ‘Residential’ classes. The least important ‘Residential’ class, which is used to connect housing within the residential areas [35], was excluded from our study, because people in living quarters navigate mainly based on subjective memory rather than spatial configurations. Then, using the suggested six classes of street data, multiple results of street network patterns were provided after conducting sDNA, such as Network Quantity Penalized by Distance in Radius Angular (NQPDA) and Line Connectivity (LConn). Here, intelligibility values are the coefficient of determination between LConn and NQPDA, ranging from 0 to 1 [36]. NQPDA value can be a proxy for urban spatial accessibility that refers to the ease with which people can reach their destinations from origins, with higher values indicating higher urban accessibility [37]. LConn measures the local spatial connectivity with higher values indicating more connected local spaces [29]. Higher intelligibility values indicate the more consistent spatial configurations between the locals and the entire city. When the intelligibility value approaches 1, local places and cities or regions are accessible and well-connected synchronically for spatial co-developments.

### 2.4 Measuring spatial configurations of local climate zones

#### 2.4.1 LCZ classification for characterizing regional spaces.

**Fig 3:**
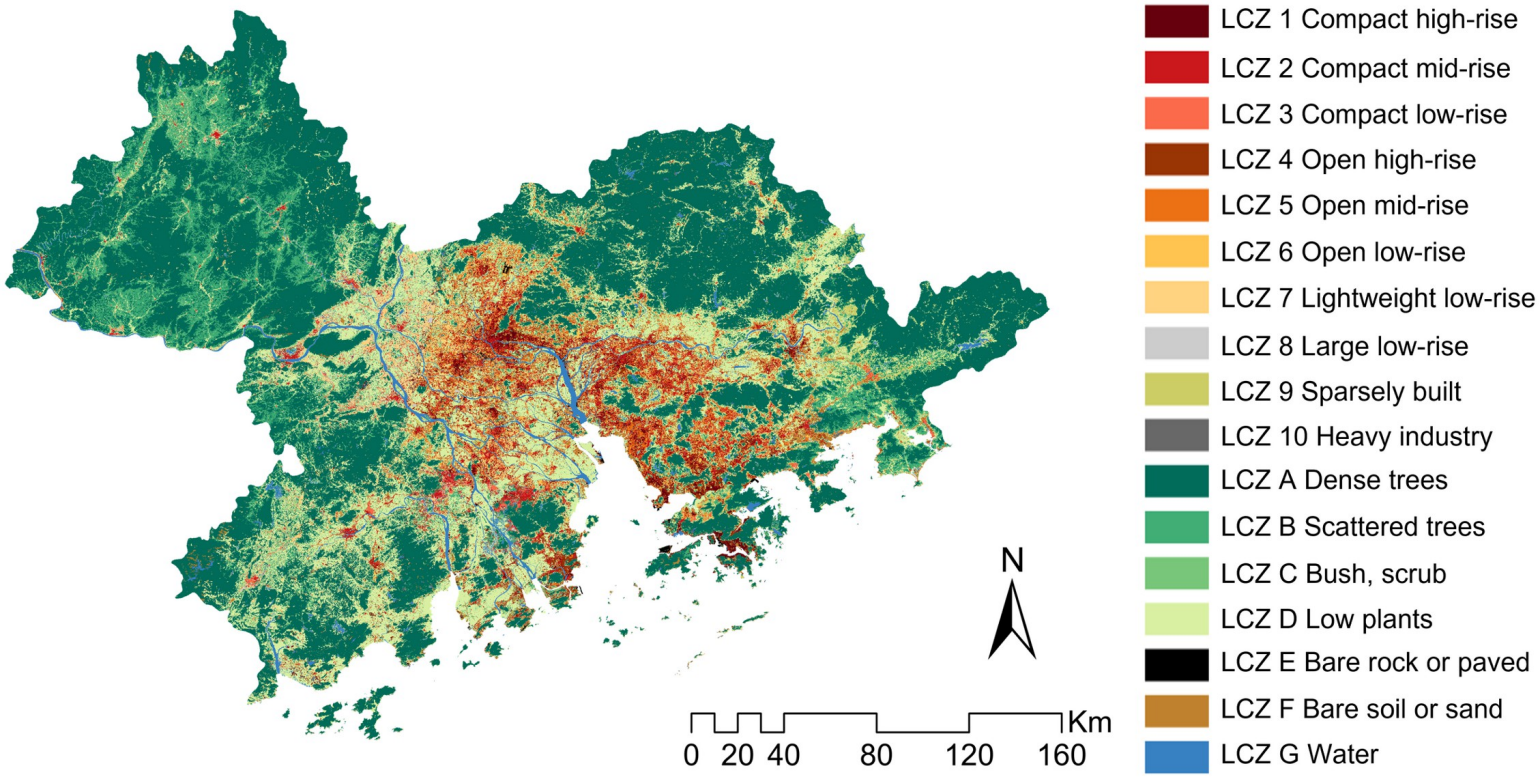
The local climate zone (LCZ) classification in the GBA in 2019.

LCZ classification is used to illustrate urban forms including land-cover types and structures. The spatial characteristic of each LCZ type has been illustrated in [19, 38]. [39] formalized a commonly used method that includes three steps: 1) preparing the Landsat data; 2) identifying training areas for LCZ classification in the SAGA software package [40], and 3) accuracy assessment and improvement. Compared to this conventional but complicated approach, a LCZ generator as a web application has been developed [38] to simplify the process, which provides not only an online platform to illustrate the urban forms of regions of interest as the LCZ maps but also an automated accuracy evaluation. Using this LCZ generator, the only thing we need to do in the LCZ classification is to define the valid training areas in the region. Training areas are the regions greater than 1 km^2^ that are defined using Google Earth^™^ to exemplify each LCZ type provided by [19]. Between 5 to 15 training samples are required according to the classification guidelines, and more training areas are preferred for improving the LCZ classification accuracy. Here, we defined 20 training areas for each LCZ type, serving as the inputs of the LCZ generator (https://lcz-generator.rub.de). The spatially explicit LCZ types in the GBA were presented in Fig 3. Its overall accuracy has been higher than most LCZ classifications in Chinese cities [41] and can also pass the quality control using international standards [42].

#### 2.4.2 Landscape metric as a proxy for quantitative LCZs configurations

Following the LCZ classification to characterize regional spaces, landscape metrics of each LCZ type are calculated to indicate urban spatial configurations, such as where a particular LCZ type is more clustered. The landscape metrics quantifications were performed using FRAGSTATS tool which is simple to use and does not require any mathematical knowledge [43]. Two levels of landscape metrics were provided in FRAGSTATS—class-level and patch-level. Patch-level metrics quantify the spatial configurations of individual patches that are the continuous areas with the homogeneous LCZ type by assigning a unique numeric value to each patch [44, 45]. Class-level metrics suggest the overall spatial configurations of LCZ types by averaging over all patch-level metrics of the corresponding LCZ type [46]. Twelve class-level and two patch-level metrics were selected, and their details were given in Table 1. The potential qualitative effects of changing landscape metrics values on wayfinding were also described in Table 1.

**Table 1:** The selected landscape metrics, their descriptions, and potentially qualitative impacts on wayfinding.

Patch-level landscape metrics			
Metrics	Abbreviation	Description	Potentially qualitative impacts on wayfinding
Euclidean nearest-neighbor distance	ENN	The shortest straight-line distance between a patch and its closest neighbor.	Decreased ENN value may improve wayfinding.
Similarity index	SIMI	The similarity between adjacent patches in terms of their land property. E.g., grasslands have a higher similarity to trees than the impervious surface.	Increased SIMI value may improve wayfinding.
Class-level landscape metrics			
Metrics	Abbreviation	Description	Potentially qualitative impacts on wayfinding
Interspersion & Juxtaposition Index	IJI	The extent to which a class intersperses and intermixes with other classes.	Increased IJI value may improve wayfinding.
Percentage of Like Adjacencies	PLADJ	The frequency with which patches of two different classes are found side-by-side in a region.	Increased PLADJ value may improve wayfinding.
Aggregation Index	AI	The extent to which two patches share an edge.	Increased AI value may improve wayfinding.
Clumpiness Index	CLUMPY	The extent to which classes are clumped. E.g., classes are maximally disaggregated when CLUMPY equals -1, distributed randomly when CLUMPY equals 0, and maximally aggregated when CLUMPY equals 1.	Increased CLUMPY value may improve wayfinding.
Landscape Shape Index	LSI	The degree to which a class is irregular in comparison to a single square patch.	Increased LSI value may improve wayfinding.
Normalized Landscape Shape Index	nLSI	The normalized version of the landscape shape index (LSI).	Increased nLSI value may improve wayfinding.
Patch Cohesion Index	COHESION	The extent to which the patches of the same class are spatially connected.	Increased COHESION value may improve wayfinding.
Number of Patches	NP	The description of the number of patches in a class.	Increased NP value may improve wayfinding.
Patch Density	PD	The number of patches of a class per unit area.	Increased PD value may improve wayfinding.
Landscape Division Index	DIVISION	The possibility of two randomly chosen patches from different classes.	Increased DIVISION value may improve wayfinding.
Splitting Index	SPLIT	The total landscape area (m^2^) is divided by the sum of patch area (m^2^).	Increased SPLIT value may improve wayfinding.
Effective Mesh Size	MESH	Perfectly inversely correlated with DIVISION.	Decreased MESH value may improve wayfinding.

### 2.5 Determinations of strategies and locations for spatial reconfigurations

#### 2.5.1 Correlation analysis

In this study, correlation analysis is used to demonstrate the associations between LCZs configurations and wayfinding. A correlation coefficient approaching +1 indicates that landscape metrics of LCZ types (proxies for LCZs configurations) and intelligibility (a proxy for wayfinding) are correlated in a more positive way, which, otherwise, are correlated in a more negative manner when the correlation coefficient approaches to -1. As a result, the correlation analysis results can be used to suggest solutions to spatial reconfigurations for wayfinding improvement.

#### 2.5.2 Hot spot analysis

The hot spot analysis helps to examine whether local spatial associations, shown as spatial clusters, exist in the urban forms presented by LCZ configurations. We used Getis-Ord Gi* [47] in the ArcGIS Pro software to conduct hot spot analysis using 90%, 95%, and 99% confidence intervals corresponding to 0.1, 0.05, and 0.01 levels of significance. Getis-Ord Gi* results with a 99% confidence interval indicate extreme clusters of hot or cold values. Hot spots are the regions where the landscape metrics with distinctly high values aggregate locally compared with surroundings, while cold spots show extremely low values of landscape metrics in statistics. Those hot or cold spots were identified as the priorities for spatial reconfigurations after determining spatial planning strategies based on correlation analysis.

## 3. Results

### 3.1 Quantifications and comparisons of wayfinding among cities

**Fig 4:**
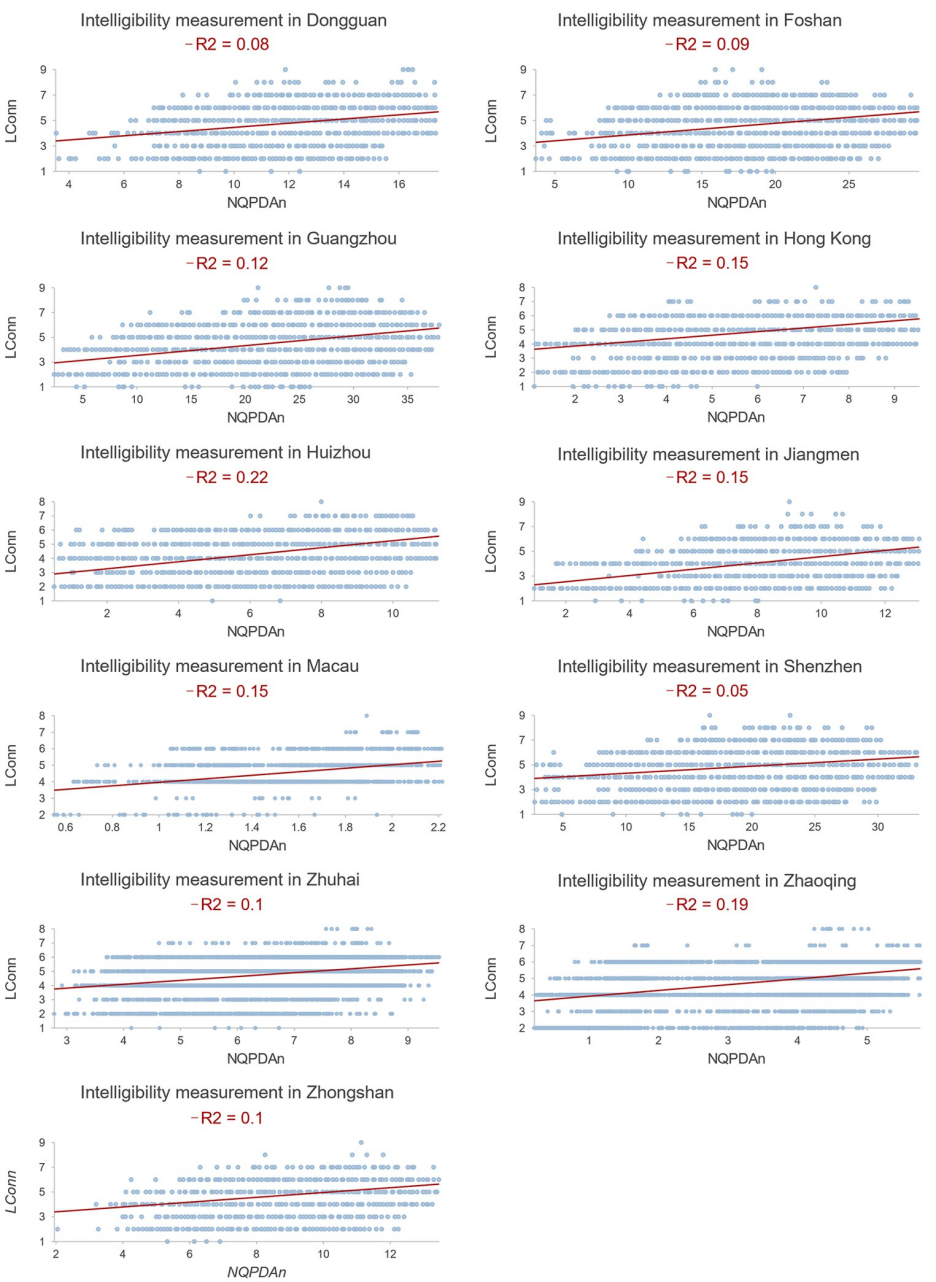
The intelligibility value to represent wayfinding in each GBA city, indicated by the R^2^ values between Lconn and NQPDA.

**Fig 5:**
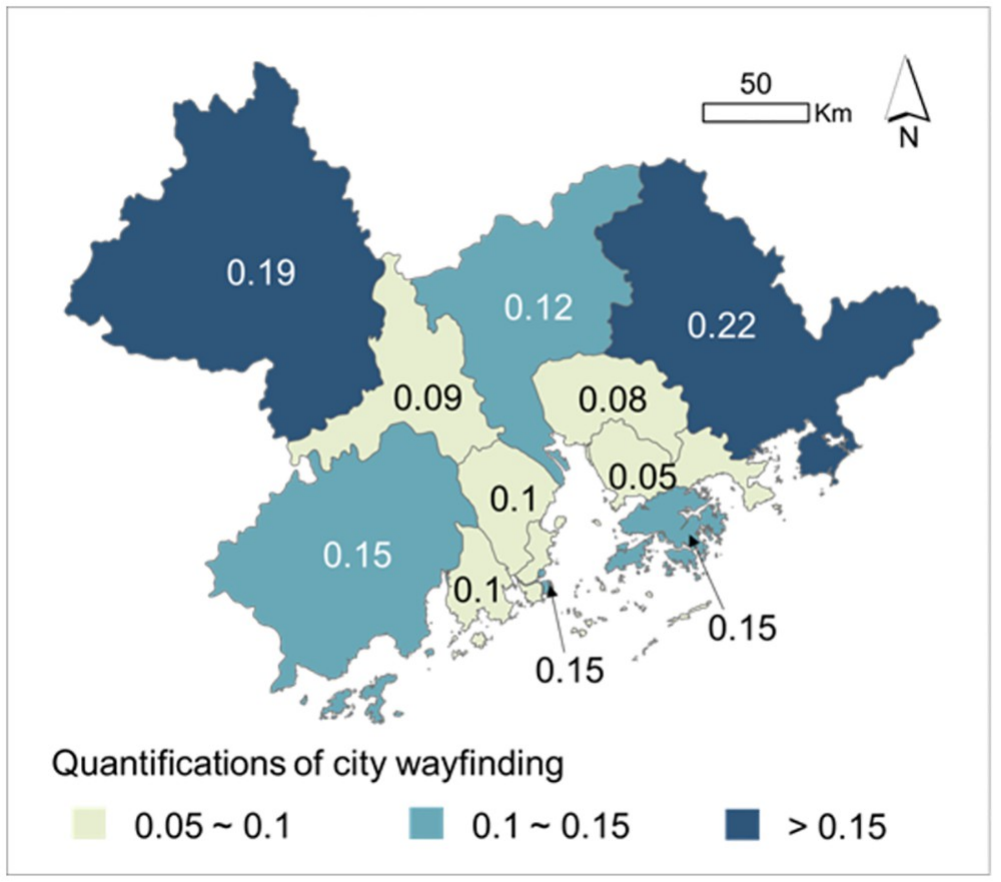
The spatial patterns of intelligibility-based wayfinding among the GBA cities.

As a proxy for wayfinding, the intelligibility value, that is, the coefficient of determination (R^2^ values) between local connectivity and city integration, has been quantified (Fig 4) to illustrate the heterogeneous wayfinding in each city (Fig 5). The whole region showed pretty low intelligibility values. Cities in the more central region, such as Shenzhen and Dongguan, had particularly lower intelligibility values than outer cities. In other words, throughout the GBA, people were hard to find their way based on the local observations of surrounding environments. This situation was worse in the regional center, because of the more asynchronous development between local connectivity and city accessibility (Fig 4). Whereas, Huizhou and Zhaoqing, as two peripheral cities in the GBA, had the highest intelligibility values, in which people find it easier to identify the spatial configurations of the whole city from the local places than people in other cities. This heterogeneity in intelligibility-based wayfinding is thought to be related to the diverse urban spatial configurations.

### 3.2 Quantitative spatial configurations in the region

The LCZ classification in the GBA has been shown in Fig 3 to illustrate the spatially heterogeneous built and land-cover types. The built-up urban forms were concentrated in the central region where the intelligibility levels were also generally lower (Fig 5), which implies the associations between wayfinding and LCZ spatial configurations. Using this LCZ map as an input of the FRAGSTATS tool, the class- and patch-level landscape metrics of each LCZ type in each GBA city can be computed.

### 3.3 Correlations of intelligibility-based wayfinding with LCZs configurations

The correlations between landscape metrics of diverse LCZ types and intelligibility values were investigated to examine whether LCZ spatial configurations have statistically significant influences on wayfinding. The results showed that, at the class level, the value of SPLIT metric in LCZ 2 (compact mid-rise) had a positive impact on intelligibility-based wayfinding with a significant level of 0.01, while negative relationships were shown between the intelligibility values and the values of PD metric in LCZ 3 (compact low-rise), LCZ 5 (open mid-rise), and LCZ 6 (open mid-rise) as well as the value of IJI metric in LCZ 9 (sparsely built) and LCZ D (low plants) (Table 2(a)). Furthermore, at the patch level, intelligibility-based wayfinding was positively affected by the value of ENN metric in LCZ1 (compact high-rise), LCZ 2 (compact mid-rise), and LCZ D (low plants) as well as the value of SIMI metric in LCZ 9 (sparsely built) (Table 2(b)).

**Table 2:** The correlations between intelligibility and landscape metrics of LCZ types.

(a) Class level						
	LCZ 2	LCZ 3	LCZ 5	LCZ 6	LCZ 9	LCZ D
City intelligibility	SPLIT	PD	PD	PD	IJI	IJI
p-values (< 0.01)	0.0087	0.00046	0.00086	0.0027	0.0052	0.0065
r	0.74	-0.87	-0.85	-0.81	-0.77	-0.76
(b) Patch level						
	LCZ 1		LCZ 2	LCZ 9		LCZ D
City intelligibility	ENN		ENN	SIMI		ENN
p-value (< 0.01)	0.0054		0.00042	0.007		0.0094
r	0.77		0.87	0.76		0.74

### 3.4 Identification of priority locations for spatial reconfigurations

The landscape metrics of LCZ types that significantly correlate with wayfinding in statistics have been identified (Table 2). Defining the current status of these metrics in each regional city is instrumental in determining the empirical strategies for spatial reconfiguration in land use. Hot spot analysis revealed the spatial clusters of those landscape metrics having statistical significance with wayfinding, indicating the cities with significantly different spatial configurations than others.

**Fig 6:**
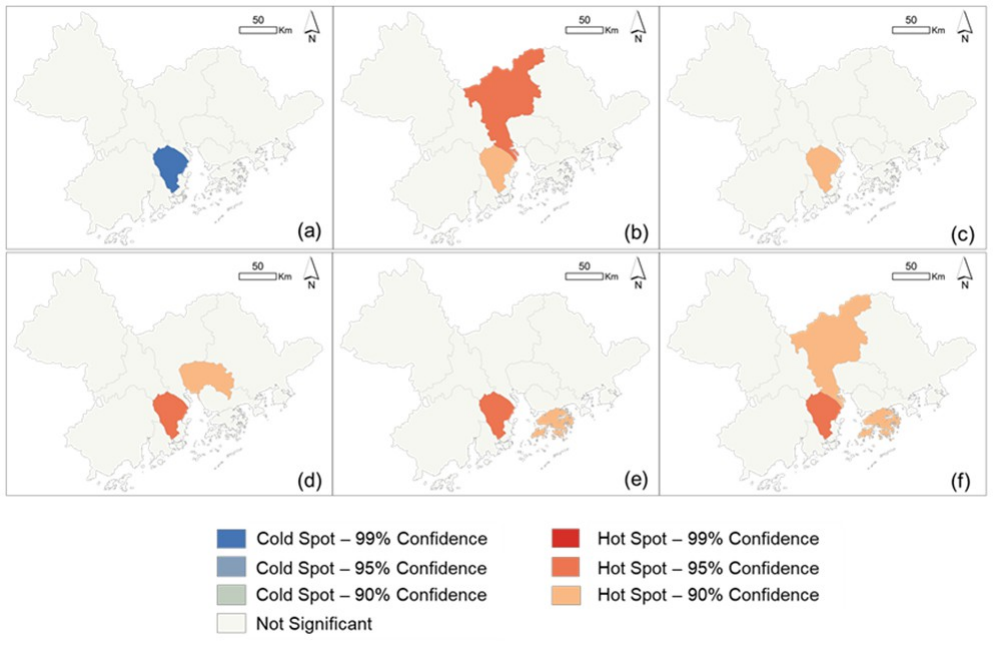
Hotspot results of class-level metrics in LCZ types. The spatial clusters of the values of SPLIT in LCZ 2 (a), the values of PD in LCZ 3 (b), LCZ 5 (c), and LCZ 6 (d), as well as the values of IJI in LCZ 9 (e) and LCZ D (f) among cities.

For class-level metrics of SPLIT, PD, and IJI, their spatial clusters among cities were presented in Fig 6. The cold spot of SPLIT values of LCZ 2 (compact mid-rise) was demonstrated in Zhongshan city (Fig 6(a)) where the LCZ 2 type was substantially aggregated due to its significantly lowest value of SPLIT compared to neighboring cities. At the same time, Zhongshan city was also the hot spots of PD values in LCZ 3 (compact low-rise), LCZ 5 (open mid-rise), and LCZ 6 (open mid-rise) as well as the hot spots of IJI values in LCZ 9 (sparsely built) and LCZ D (low plants) (Fig 6(b)–6(f)), which means that LCZ 3, LCZ 5, and LCZ 6 types in Zhongshan city were significantly subdivided than other cities, and LCZ 9 and LCZ D types were highly interspersed into other LCZ types. Moreover, LCZ 3 and LCZ D types were both particularly disaggregated in Guangzhou city shown as the hotspots of both PD and IJI (Fig 6(b), 6(f)). Dongguan city also had separately distributed LCZ 6, similar to Zhongshan city (Fig 6(d)). LCZ 9 and LCZ D types were highly juxtaposed with other LCZ types in Hong Kong (Fig 6(f)).

**Fig 7:**
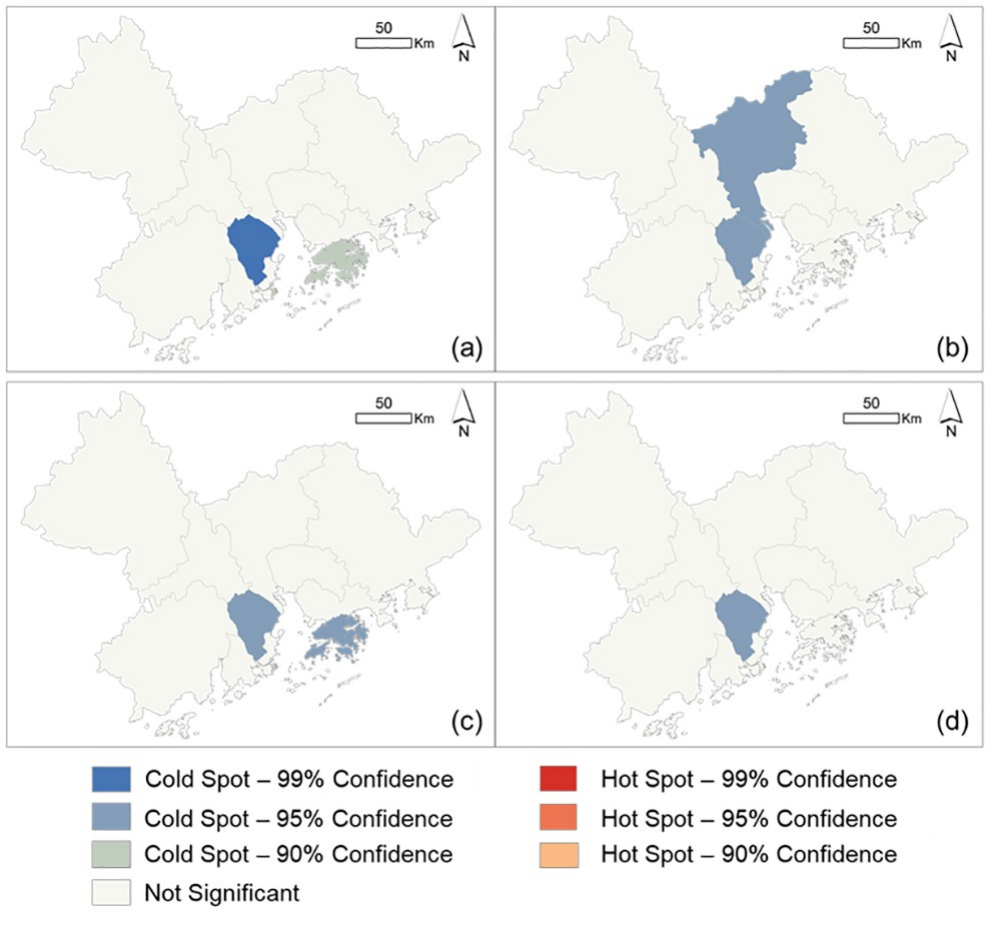
Hotspot results of patch-level metrics in LCZ types. The spatial clusters of the values of ENN in LCZ 1 (a) and LCZ 2 (b), the values of SIMI in LCZ 9 (c), and the values of ENN in LCZ D (d) among cities.

For patch-level metrics of ENN and SIMI, Zhongshan city was still the cold spot of all patch-level landscape metrics, in which patches in LCZ1 (compact high-rise), LCZ 2 (compact mid-rise), and LCZ D (low plants) were more spatially connected due to their significantly shorter interpatch distances, compared to other cities (Fig 7(a), 7(b) and 7(d)). Patches of LCZ 9 type exhibited the least similarity to their adjacencies in Zhongshan and Hong Kong (Fig 7(c)). Comparing the spatial clusters of LCZs between class and patch levels, LCZ 9 showed the same patterns among cities (Figs 6 and 7(c)), whereas a contrast configuration of LCZ D type between class and patch levels was observed (Figs 6(f) and 7(d)). In detail, LCZ D type was overall intermixed with other LCZ types on average, and the patches of LCZ D type were contrarily spatially clustered.

Overall, hot spot analysis for landscape metrics contributed to determining the priority cities for spatial reconfigurations. The locations showing hot or cold spots had significantly different landscape metrics of LCZ types, in which spatial reconfigurations had the potential to improve wayfinding greatly. In our case, spatial clusters of landscape metrics were shown in Zhongshan, Guangzhou, Dongguan, and Hong Kong. Here, we defined Zhongshan as the priority location for spatial reconfigurations, because Zhongshan is the only city that shows hot or cold spots of each landscape metric (Figs 6 and 7). The details about the spatial distributions of LCZ types can refer to S1 Fig in S1 File.

## 4. Discussion

Explaining intelligibility-based wayfinding using spatial analysis of urban forms, this study provides not only an insight into understanding wayfinding as a social issue from the perspective of physical spatial characteristics but also empirical suggestions for regional spatial planning via LCZ reconfigurations. The effects of scale in landscape metrics on spatial configurations quantifications and spatial planning solutions are also demonstrated.

### 4.1 Understanding wayfinding socially using spatial characteristics physically

#### 4.1.1 Intelligibility-based measurements of wayfinding based on spatial coherence

Wayfinding, based on its definition, reflects the cognitive benefits derived from physical urban spaces in human movement. In environmental psychology, wayfinding is commonly seen as a subjective aspect of human walking. Using intelligibility measurement, this study offers a spatial configuration perspective to understand and explain the wayfinding while walking, by defining the spatial coherence between the local and the entire city. When people cognize more coherent and consistent spatial knowledge in the process of travelling through diverse urban spaces, they have a clear ‘big picture’ of the entire urban spatial configurations easily, and then their walking needs in terms of wayfinding will be better met. Empirically, intelligibility is quantified by the correlation between local connectivity and city accessibility [8, 48]. Both city accessibility and local connectivity exert physical demands on intelligibility-based wayfinding in human travel [49]. Planners have been motivated to improve accessibility and connectivity in urban spatial or transport planning for encouraging human physical activities [50]. However, improving accessibility and connectivity separately may not necessarily create an easy wayfinding urban form. In our case, Foshan, Guangzhou, Dongguan, and Shenzhen have the greatest accessibility throughout the city (Fig 4), because of their socioeconomic function in the whole region. However, their lower local connectivity (Fig 4) causes lower intelligibility values and a weaker ability for wayfinding than other cities (Fig 5). Interestingly, in Huizhou and Zhaoqing, which have the highest intelligibility values, both local connectivity and city accessibility are low (Fig 4). Therefore, better wayfinding does not always imply improved local connectivity or city accessibility, vice versa. Instead, it simply indicates the extent of spatial coherence between local and city levels. Future spatial planning should emphasize the synchronous pattern of improving local and urban spatial configuration for improving social wayfinding through physically spatial co-development at the local, urban, and regional levels.

#### 4.1.2 Empirical associations between spatial configuration and wayfinding

Empirical evidence in this study revealed significant correlations between landscape metrics of LCZ types and intelligibility values (Table 2), achieving the research objective of defining statistical relationships between intelligibility-based wayfinding and LCZ-based urban forms. Thus, we claim that the wayfinding in human movement can be improved by adjusting configurations of LCZ types. The issue of wayfinding in human-related social science is then transformed into the issue of configurational changes in space-related urban science.

Our claim is also supported by several previous studies that have detected the influences of spatial information cognized during the trip on wayfinding or spatial orientation [51]. Spatial layouts at both local and city levels, such as spatial symmetry or complexity, were considered important determinants of human walking behavior, spatial knowledge acquisition, and then the level of wayfinding [12, 52]. [10] proposed a model to demonstrate how the spatial geometry of street corners affects wayfinding based on the 532 sets of surveys from 20 participants. [53] found that regular geometry in an urban setting can increase intelligibility and thus wayfinding based on stimulation.

Identifying statistically significant relationships between spatial configuration and wayfinding, this study is unique in that it investigated the spatial configuration impacts of different LCZ-based urban forms. In other words, rather than focusing on general spatial layouts without taking into account urban function and land use, we provide further details about the impacts of various LCZ types, allowing for more precise spatial adjustments in practice. We not only complement the literature by pointing out the direction and magnitude of correlations between spatial configurations and wayfinding but also show how spatial planning strategies can be implemented.

### 4.2 Scale affects landscape metrics quantifications

Landscape metrics quantification is scale-dependent [54], which means that the numeric values as representations of spatial configurations within a city or a region may differ according to the spatial scale of the land data. Scale, in the context of landscape metrics, generally includes the ‘extent’ and ‘grain’ [55]. Extent is the geographical scope, which refers to the boundary of the GBA in this study; Grain refers to the spatial resolution of the observation units [56]. Scale effects then occur when quantifying landscape metrics-based spatial configurations. Firstly, landscape metrics of LCZs may change when choosing each GBA city as the extent instead of the entire regional extent. Moreover, the grain of patch-level landscape metrics depends on the spatial resolution of the basic land data. In this study, the grain of spatial configurations of individual LCZ patches is the same as the spatial resolution of LCZ classification, which is 100*100 m^2^. However, it may vary after changing the data resolution. For example, [57] used vegetation raster data with a grain of 20*20 cm^2^ for measuring spatial configuration. Meantime, focusing on the overall LCZ configurations, the observation unit shifts from LCZ patch to the LCZ type, and then the coarser grain of class-level landscape metrics may suggest results that differ from patch-level landscape metrics. The opposite effects of LCZ D (low plants) on wayfinding between patch- and class-level landscape metrics (Table 2) support this statement. To improve wayfinding, a more clustered spatial layout of the overall LCZ D is recommended based on the class-level landscape metrics, whereas patch-level results suggest scattered distributions of LCZ D patches (Table 2). This contrast requires some supplementary analysis methods, such as social surveys and fieldwork.

### 4.3 Spatial reconfigurations strategies for improving wayfinding

#### 4.3.1 Dispersed compact high- and mid-rise built-ups

Higher values of ENN and SPLIT have a positive impact on intelligibility-based wayfinding, in LCZ 1 (compact high-rise) and LCZ 2 (compact mid-rise), respectively (Table 2). Thus, for a better wayfinding ability, we suggest increasing the straight-line distance between LCZ 1 or subdividing LCZ 2 into smaller entities, as shown in Fig 7(a) and 7(b). From an urban function perspective, compact high- and mid-rise built-up areas (LCZ 1 and LCZ 2), as commercial or business districts, are generally located in the city centers and usually have great accessibility. However, LCZ 1 and LCZ 2 may not connect to surrounding spaces well, because more central regions possibly have different spatial characteristics than other built-up types (e.g., distant residential districts). Low local connectivity but high accessibility in LCZ 1 and LCZ 2 causes lower intelligibility levels and thereby increased difficulties in wayfinding. According to our empirical results, more dispersed distributions of LCZ 1 and LCZ 2 types are reasonable.

#### 4.3.2 Agglomerated compact low-rise, open mid- and low-rise built-ups

**Fig 8:**
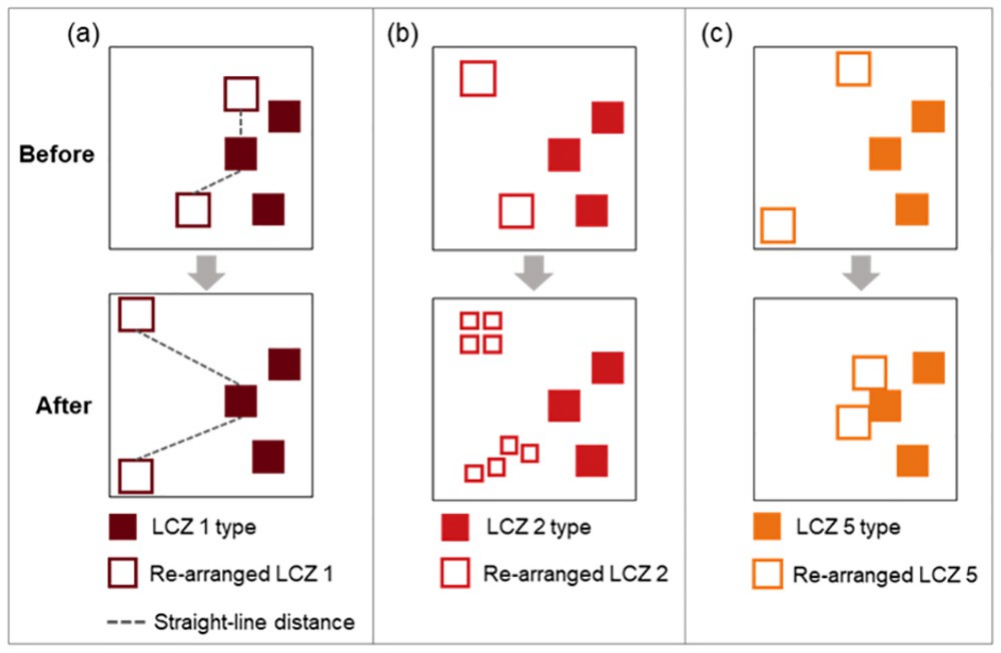
Diagrams to illustrate the spatial reconfigurations of LCZ 1(a), LCZ 2 (b), and LCZ 5 (c).

In LCZ 3 (compact low-rise), LCZ 5 (open mid-rise), and LCZ 6 (open low-rise) types, PD values are negatively correlated to intelligibility-based wayfinding (Table 2), which means that more agglomerated LCZ 3, LCZ 5, and LCZ 6 are desirable by combining and connecting the individual small LCZ patches into several larger-scale units. An example of agglomerating LCZ 5 has been illustrated in Fig 8. The open built-up areas (LCZ5 and LCZ 6) generally have abundant trees or green-spaces [19] and thus have greater economic values particularly in the high-density regions. Higher-income cohorts may occupy the majority of LCZ 5 and LCZ 6, resulting in spatial segregation, gentrification, and lower accessibility. Spatial segregation causes disparities in spatial configuration and, as a result, poor wayfinding. Moreover, ‘urban village’ is typical of LCZ 3 (compact low-rise), as an intermediate urban form in the urbanization process [58], and has an isolated spatial configuration from the rest of the urban spaces. Thus, people around these urban villages are more likely to become disoriented and find it difficult to travel effortlessly by accurately positioning themselves within the city. Therefore, the suggestion of more aggregated LCZ 3, LCZ 5, and LCZ 6 types allows for less spatial segregation, better spatial co-developments, and thus better wayfinding.

## 5. Conclusion

To enable the formulation of spatial reconfigurations strategies for improving wayfinding, our study provides empirical results demonstrating wayfinding performance using an intelligibility indicator, quantifies the spatial configurations of LCZ types using landscape metrics, and investigates their associations quantitively. Our study is novel in that it is the first study, to our knowledge, that applies LCZ classification to an intelligibility-based wayfinding methodology in the field of spatial planning. Another contribution of this research is the empirical evidence for suggesting strategies for future spatial reconfigurations, based on the statistical relationships between intelligibility values and multi-level landscape metrics of urban form.

The main results show, firstly, the overall poor wayfinding performance in the whole region with the particularly low intelligibility values in more central cities, in which, people have greater difficulty in orienting themselves and finding their ways within complex urban spaces, because of the incoherent local and urban spatial configurations. Secondly, the correlations between landscape metrics of LCZ types and intelligibility-based wayfinding are statistically significant. This correlation analysis provides empirical evidence for spatial reconfiguration strategies for future urban and regional planning. In general, we claim that dispersed compact built-up areas and agglomerated non-high-rise built-up areas are desired in the region. Specifically, we suggest 1) increasing the distance between LCZ 1 (compact high-rise) and LCZ 2 (compact mid-rise) for their more dispersed distribution; and 2) agglomerating LCZ 3 (compact low-rise), LCZ 5 (open mid-rise), LCZ 6 (open low-rise), and LCZ 9 (sparsely built) for their more clustered distributions. In addition, Guangzhou, Dongguan, and Hong Kong, and particularly Zhongshan are defined as the priority locations for spatial reconfigurations based on the spatial clusters of landscape metrics.

Although the accuracy of LCZ classification in characterizing urban form needs to be improved further, the various intelligibility-based wayfinding in different cities has been explained by the spatial analyses of urban forms. We show not only the development of specific strategies for configurational changes in the region corresponding to diverse LCZ types but also the identification of priority locations for spatial reconfiguration implementation. Understanding wayfinding as a social issue from the perspective of physical spatial configurations contributes to developing human-centered urban and regional spaces, through the synchronous local and urban spatial configurations for co-development at local, city, and region levels.

## Supporting information

S1 File Zhongshan city is defined as a priority location for spatial reconfiguration in this study. Its spatial distributions of LCZ types are shown at S1 Fig.
(DOCX)
