# Supplementary material for: The spatial configuration of local climate zones reveals effects on wayfinding in human walking
Source: PLoS One. 2023 Sep 8;18(9):e0289780. doi: 10.1371/journal.pone.0289780 (PMC10490918; doi:10.1371/journal.pone.0289780)
Supplement: S1 File — Its spatial distributions of LCZ types are shown at S1 Fig. (DOCX) [file pone.0289780.s001.docx]

**Supporting Information**

Focusing on the Zhongshan city, the spatial distributions of seven LCZ types that significantly correlated to intelligibility-based wayfinding were presented in S1 Fig. In detail, the regions of LCZ 1 and LCZ 2 (shown by polygon in red in S1(a1-a2)) Fig were suggested to be distant by increasing their straight-line distance. The number of individual patches of LCZ 3, LCZ 5, and LCZ 6 types within the city should be reduced by connecting and aggregating them (S1(a3-a5) Fig). Individual LCZ 9 patch was also recommended to be clustered for increasing the land surface similarity between LCZ 9 and its neighboring LCZ types (S1(a6) Fig). Apart from general spatial reconfiguration strategies for the entire regional wayfinding consideration, the priority city defined by hot spot analysis provided clues for more specific city-level spatial planning.


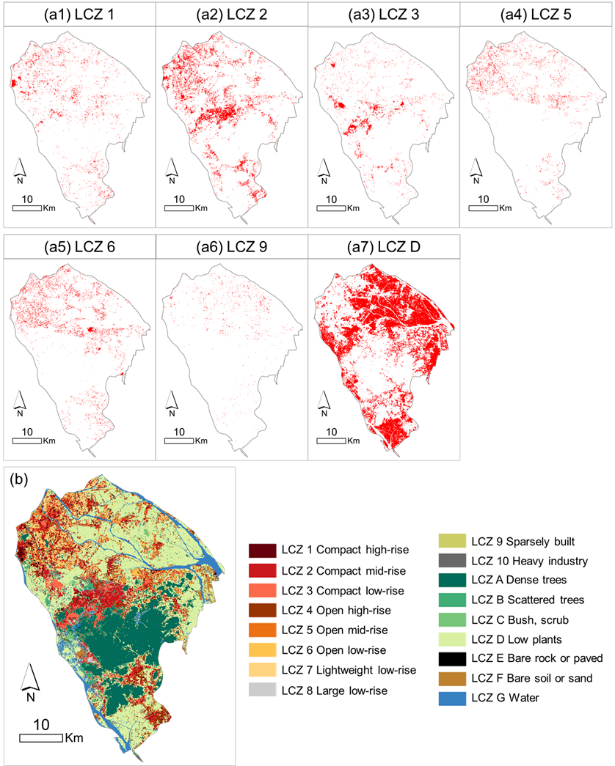


**S1 Fig. Zhongshan as a priority location for spatial reconfiguration.** The spatial distributions of seven LCZ types that are significantly correlated with wayfinding in Zhongshan city are shown in (a1-a7). The whole city LCZ classifications are also shown in (b).
